# Knowledge and Attitudes Towards Topical Corticosteroids Among Previous Users in the General Population of Saudi Arabia

**DOI:** 10.7759/cureus.55373

**Published:** 2024-03-02

**Authors:** Raham A Alamri, Hanadi S Al Satti

**Affiliations:** 1 Department of Dermatology, King Abdulaziz Medical City, Jeddah, SAU

**Keywords:** saudi arabia, medications misuse, topical corticosteroid phobia, public knowledge, public awareness, topical corticosteroid

## Abstract

Introduction

Topical corticosteroids are a valuable tool for treating many dermatoses, offering anti-inflammatory and immunosuppressive properties. However, preexisting knowledge gaps and safety concerns may hinder treatment compliance. This study aims to evaluate knowledge and attitudes towards topical corticosteroids among former users within the general population of Saudi Arabia.

Methods

This cross-sectional study utilized an online survey to collect data. Knowledge was assessed through three dimensions: indications, proper use, and adverse reactions. Attitudes were assessed using the Topical Corticosteroid Phobia (TOPICOP) scale.

Results

Among the 397 respondents, 80.9% were females, 51.1% had suffered from a dermatological disease, and 76.3% had a bachelor's or higher educational level. When assessing knowledge, female participants (6.22±2.02) displayed significantly higher scores compared to male participants (5.26 ± 2.23) (p<0.001). Participants with dermatological diseases provided more accurate answers compared to those without. In assessing phobia towards topical corticosteroids, participants aged 18-25 years had lower topical corticosteroid phobia scores (31.06 ± 5.91), whereas those aged 56 years or more had higher scores (35.38 ± 6.04), p<0.001. Single participants had significantly lower topical corticosteroid phobia scores (32.27 ± 6.06) compared to those who were married (33.87 ±5.95) (p=0.010). Additionally, participants with dermatological diseases had higher scores in the behaviors subcategory despite having lower Global TOPICOP scores (32.58 ± 5.7) (p=0.033).

Conclusion

Enhancing knowledge about topical corticosteroids is crucial for mitigating corticophobia and promoting better adherence. To address gaps in knowledge, dermatologists should expand educational initiatives to include vulnerable populations, explicitly targeting males and older individuals.

## Introduction

Topical corticosteroids are a valuable tool for treating many dermatoses through their anti-inflammatory and immunosuppressive properties. Examples of these dermatoses include psoriasis, lichen sclerosis, vitiligo, atopic dermatitis (AD), and other forms of eczema. Successful management relies on an accurate diagnosis and consideration of the vehicle, potency, frequency of application, and adverse reactions of the corticosteroids. Reported adverse reactions include skin atrophy, rosacea, peri-oral dermatitis, acne, and systemic absorption [1]. Furthermore, information regarding the potential hazards of potent topical corticosteroids has been widely disseminated, leading to misinformation and topical corticophobia.

Many studies have evaluated the implications of misinformation concerning topical corticosteroids on patients and healthcare professionals (HCPs). In a cross-sectional survey conducted in Riyadh, Saudi Arabia, 80.8% of patients with dermatological diseases expressed topical corticophobia to varying degrees. Most of these fears concern the safety profile of topical corticosteroids, leading to poor therapeutic adherence and, consequently, insufficient treatment response [2]. Another study in Denmark demonstrated a significant association between topical corticophobia and low educational levels among parents of children with AD, resulting in delayed treatment of AD flares [3]. Conversely, caretakers of children with AD in Japan expressed topical corticophobia regardless of their background [4]. Furthermore, knowledge gaps towards topical corticosteroids among HCPs could further hinder treatment response and amplify possible adverse reactions. In a survey conducted among primary care physicians in Riyadh, responders demonstrated inadequate knowledge regarding the potency classification of selected topical corticosteroid agents. For instance, 81% of responders failed to recognize clobetasol propionate as ultra-high potency, whereas 44.3% reported prescribing it [5]. Likewise, Australian pharmacists had poor knowledge of topical corticosteroids' indications and safety profile [6]. Both studies proposed targeted education delivered by dermatologists as a solution. Although topical corticosteroids are widely prescribed, no studies examine the knowledge and attitudes among different demographics of the general population in Saudi Arabia.

The aim of this study is to assess the knowledge and attitudes towards topical corticosteroids among a sample of previous users within the general population in Saudi Arabia. It is particularly significant to collect local data to provide a thorough understanding of the public perception of commonly prescribed medications, identify obstacles to efficient treatment, and overcome them. Furthermore, the data collected could aid in identifying areas with gaps in knowledge and the demographics at most risk, as well as modify current patient education practices.

## Materials and methods

Study design and population

This cross-sectional study collected data through an online survey distributed via social media. When assessing responses to evaluate knowledge and phobia towards topical corticosteroids, the inclusion criteria included males and females, aged 18 years or older, who were previous users from different socio-demographic backgrounds. The exclusion criteria were participants who had not previously used topical corticosteroids. Convenience sampling was utilized.

Study setting, procedure, and validation

The research was conducted online, using social media platforms to distribute the survey. This virtual approach enabled broad participation, reaching individuals from diverse demographics across Saudi Arabia. The survey was divided into two sections. The first section assessed knowledge about topical corticosteroids through three dimensions: indications versus contraindications, proper use, and adverse reactions. The questions for this part were developed by the authors of this study and validated by five expert dermatologists and an independent biostatistician to ensure the reliability of the survey. The second section evaluated attitudes towards corticosteroids using the TOPICOP scale. TOPICOP is a validated survey consisting of 12 items related to "worries" (6 items) and "beliefs" (6 items). The "worries" items were further divided into "fears" and "behaviours" [7].

Statistical analysis

Using the Raosoft sample size calculator, with a 5% margin of error and an estimated population size of 20,000 and above, the minimum required sample size was determined to be 377. Statistical analysis was performed using SPSS version 23. Descriptive statistics, including frequencies and percentages, were used for continuous data. A comparison of total knowledge scores and attitude scores between different categorical variables was conducted using the T-test and ANOVA. All statistical analyses were two-sided, with a significance value (α) of p<0.05 considered statistically significant, and the power of the test (β) was set at 80%.

## Results

Participants' characteristics

A total of 397 responses were included in the study. Of these participants, 321 (80.9%) were females, 118 (29.7%) belonged to the age group of 18-25 years, 364 (91.7%) were Saudi citizens, 204 (51.4%) were from the Western province of Saudi Arabia, 303 (76.3%) had a bachelor's or higher educational level, and 238 (59.4%) were married (Table 1).

**Table 1:** Profile of study participants.

Demographic variables		Frequency	Percentage
Gender	Female	321	80.9
	Male	76	19.1
Age in Years	18-25	118	29.7
	26-35	94	23.7
	36-45	107	27.0
	46-55	64	16.1
	>=56	14	3.5
Nationality	Saudi	364	91.7
	Non-Saudi	33	8.3
Region of Residence	Central	71	17.9
	Eastern	37	9.3
	Northern	24	6.0
	Southern	61	15.4
	Western	204	51.4
Educational Level	Primary	2	.5
	Intermediate	12	3.0
	Secondary	80	20.2
	Bachelors and above	303	76.3
Marital Status	Single	161	40.6
	Married	236	59.4

Practices related to topical corticosteroid use

According to the results in Table 2, 389 (98%) of participants had previously used topical corticosteroids, and about 51.1% had suffered from dermatological diseases (p=0.004). The most common indication for use was allergies, urticaria, or irritation (33.7%), followed by AD or eczema (21.6%), hyperpigmentation (10%), and chemical peeling (4.9%). Among the 199 (51.2%) participants who suffered from a dermatological issue, the most common condition was AD or eczema (42.2%), followed by acne (16.6%) and hyperpigmentation (10.1%). It was reported by 154 (38.8%) of the participants that topical corticosteroids were unsafe, and no significant differences were observed between individuals with dermatological issues and those without (p=0.968). The most common source of information about topical corticosteroids overall was physicians (44.8%), followed by social media platforms (19.1%), family and friends (15.6%), and online articles (10.8%). Physicians were the most common source among those with a dermatological disease, while other sources such as pharmacists, family and friends, and social media platforms were more common among individuals without dermatological diseases (p=0.002).

**Table 2:** Practices and perceptions of topical corticosteroid. * Only previous users of topical corticosteroids were included (N=389). P-value for chi-square test (<0.05 is considered statistically significant).

		Participants with dermatological disease		Total	P-value
		No	Yes		
Used Topical Corticosteroids Before	Yes	190	199	389	0.004
		48.8%	51.2%	100.0%	
	No	8	0	8	
		100.0%	0.0%	100.0%	
Indications of use (n=389)*	Acne	11	25	36	<0.001
		30.6%	69.4%	100.0%	
	After laser hair removal	23	9	32	
		71.9%	28.1%	100.0%	
	Allergies/Urticaria/Irritation	79	52	131	
		60.3%	39.7%	100.0%	
	Burns	9	2	11	
		81.8%	18.2%	100.0%	
	Chemical Peel	13	6	19	
		68.4%	31.6%	100.0%	
	Wound care	15	1	16	
		93.8%	6.3%	100.0%	
	Fungal infection	1	3	4	
		25.0%	75.0%	100.0%	
	Hyperpigmentation/Whitening	23	16	39	
		59.0%	41.0%	100.0%	
	Infections	0	1	1	
		0.0%	100.0%	100.0%	
	Psoriasis	0	9	9	
		0.0%	100.0%	100.0%	
	Rosacea	0	5	5	
		0.0%	100.0%	100.0%	
	Scarring	0	1	1	
		0.0%	100.0%	100.0%	
	Seborrheic Dermatitis	1	3	4	
		25.0%	75.0%	100.0%	
	Skin infection	7	1	8	
		87.5%	12.5%	100.0%	
	Skin Moisturizer	9	2	11	
		81.8%	18.2%	100.0%	
	Atopic Dermatitis/Eczema	0	84	84	
		0.0%	100.0%	100.0%	
Perception of safety of topical corticosteroids	Not safe	77	77	154	0.968
		50.0%	50.0%	100.0%	
	Safe	121	122	243	
		49.8%	50.2%	100.0%	
Source of information about topical corticosteroids	Physicians	70	108	178	0.002
		39.3%	60.7%	100.0%	
	Pharmacists	26	12	38	
		68.4%	31.6%	100.0%	
	Family & friends	37	25	62	
		59.7%	40.3%	100.0%	
	Online articles	21	22	43	
		48.8%	51.2%	100.0%	
	Social Media	44	32	76	
		57.9%	42.1%	100.0%	

Knowledge of topical corticosteroid indications, use, and adverse reactions

Knowledge was assessed among previous users (N=389) using ten items. The total scores were calculated based on participants' responses, where correct responses were given a score of '1' and incorrect responses were given no score. Half a score was assigned for those knowledge items with multiple correct responses. The maximum total knowledge score for one participant was 10, and the minimum was 0. Our analysis showed that the total knowledge score was 6.05 ± 2.09.

When comparing the total knowledge scores across different sociodemographic characteristics (Table 3), it was observed that female participants (6.22±2.02) displayed significantly higher scores compared to male participants (5.26 ± 2.23) (p<0.001). However, other characteristics such as age, nationality, marital status, region, and educational level of the participants did not show any statistically significant differences in knowledge scores (p>0.05). Participants with dermatological diseases were compared to those without (Table 4). Those with dermatological diseases answered one question significantly more accurately: "Topical Steroids Can Increase The Visibility of Blood Vessels on the Skin" (p=0.042). Also, participants with dermatological diseases mentioned 'allergies and eczema' as one of the indications for topical corticosteroids more than others (p<0.001). However, no significant differences were observed for other question responses between the two groups (p>0.05).

**Table 3:** Comparison of knowledge scores based on sociodemographic details (N=389). Only previous users of topical corticosteroids were included (N=389). P-value for student's t-test/ANOVA (<0.05 is considered statistically significant). The maximum knowledge score is 10.0, and the minimum is 0.

Demographic variables		N	Mean	SD	P-value
Gender	Female	317	6.22	2.02	<0.001
	Male	72	5.26	2.23	
Age in Years	18-25	117	6.06	2.02	0.884
	26-35	93	6.03	2.08	
	36-45	105	6.19	2.20	
	46-55	61	5.83	2.16	
	>=56	13	6.08	1.91	
Nationality	Saudi	358	6.07	2.07	0.346
	Non-Saudi	31	5.70	2.35	
Marital Status	Single	159	6.14	1.97	0.475
	Married	230	5.98	2.17	
Region	Central	67	6.26	2.01	0.682
	Eastern	37	6.28	1.85	
	Northern	24	5.85	2.41	
	Southern	60	6.18	2.10	
	Western	201	5.92	2.13	
Educational Level	Primary	2	5.50	2.12	0.319
	Intermediate	12	5.38	2.37	
	Secondary	79	5.77	2.29	
	Bachelors and above	296	6.16	2.03	
Experienced Dermatological Issue	No	190	5.88	2.13	0.127
	Yes	199	6.21	2.05	

**Table 4:** Responses related to knowledge about topical corticosteroids (N=389). * Correct responses Only previous users of topical corticosteroids were included (N=389). P-value for Chi-square test (<0.05 is considered statistically significant).

Knowledge Subcategory	Questions	Participants without dermatological disease				Participants with dermatological disease				P-value	
		True		False		True		False			
Formulations	Topical steroids can have different formulations like cream, ointment, gels, foams	156* (82.1%)		34 (17.9%)		160* (80.4%)		39 (19.6%)		0.667	
Proper use	High-potency topical steroids can be used for more than three weeks continuously	41 (21.6%)		149* (78.4%)		36 (18.1%)		163* (81.9%)		0.388	
Side effects	Topical steroids are associated with significant skin adverse effects	148* (77.9%)		42 (22.1%)		157* (78.9%)		42 (21.1%)		0.811	
	Topical steroids can cause skin thinning	112* (58.9%)		78 (41.1%)		133* (66.8%)		66 (33.2%)		0.107	
	Topical steroids can cause changes in skin pigmentation	139* (73.2%)		51 (26.8%)		152* (76.4%)		47 (23.6%)		0.464	
	Topical steroids can increase the visibility of blood vessels on the skin	112* (58.9%)		78 (41.1%)		137* (68.8%)		62 (31.2%)		0.042	
	Topical steroids can increase hair growth	76* (40.0%)		114 (60.0%)		74* (37.2%)		125 (62.8%)		0.569	
	Topical steroids can cause delayed wound healing	65* (34.2%)		125 (65.8%)		73* (36.7%)		126 (63.3%)		0.610	
	Side effects of topical steroids are irreversible	58* (30.5%)		132 (69.5%)		50* (25.1%)		149 (74.9%)		0.234	
Proper indications	Allergies & eczema	113* (59.5%)		77 (40.5%)		160* (80.4%)		39 (19.6%)		<0.001	
	Red, itchy, burning skin	93* (48.9%)		97 (51.1%)		113* (56.8%)		86 (43.2%)		0.122	
	Skin color lightening	43 (22.6%)		147* (77.4%)		39 (19.6%)		160* (80.4%)		0.463	
	Acne	43 (22.6%)		147* (77.4%)		59 (29.6%)		140* (70.4%)		0.116	
	Pigmentation	45 (23.7%)		145* (76.3%)		53 (26.6%)		146* (73.4%)		0.503	
	I don't know	29 (15.3%)		161* (84.7%)		8 (4.0%)		191* (96.0%)		<0.001	

Prevalence of topical corticosteroid phobia

The phobia towards topical corticosteroids was evaluated among previous users (N=389) using the TOPICOP scale [7]. Four response choices were offered, from totally disagree to totally agree, with points attributed to each one (1, 2, 3, or 4), with higher values indicating more severe topical corticosteroid phobia. Individual scores for all participants were calculated by summing responses to items (12 items) and then dividing that value by the number of items completed, yielding a maximum score of 48, expressed as a percentage. The analysis showed that the mean topical corticosteroid phobia score was 33.21± 6.03.

The comparison of topical corticosteroid phobia scores between different sociodemographic characteristics of the participants is presented in Table 5. It was observed that participants aged 18-25 years had lower topical corticosteroid phobia scores (31.06 ± 5.91), whereas those aged 56 years or more had higher scores (35.38 ± 6.04), p<0.001. Single participants had significantly lower topical corticosteroid phobia scores (32.27 ± 6.06) compared to those who were married (33.87 ±5.95) (p=0.010).

**Table 5:** Comparison of TOPICOP scores based on sociodemographic details (N=389). Inclusion criteria limited to previous users of topical corticosteroids (N=389). P-values derived from student's t-test/ANOVA (<0.05 is considered statistically significant). The TOPICOP score ranges from a minimum of 12 to a maximum of 48. TOPICOP: Topical Corticosteroid Phobia.

Demographic variables		N	Mean	SD	P-value
Gender	Female	317	33.44	5.96	0.113
	Male	72	32.19	6.30	
Age in Years	18-25	117	31.06	5.91	<0.001
	26-35	93	33.78	5.68	
	36-45	105	33.87	6.07	
	46-55	61	34.89	5.78	
	>=56	13	35.38	6.04	
Nationality	Saudi	358	33.16	5.95	0.548
	Non-Saudi	31	33.84	7.10	
Marital Status	Single	159	32.27	6.06	0.010
	Married	230	33.87	5.95	
Region	Central	67	33.28	5.47	0.102
	Eastern	37	33.73	5.11	
	Northern	24	34.75	5.42	
	Southern	60	34.58	5.94	
	Western	201	32.50	6.40	
Educational Level	Primary	2	30.00	9.90	0.608
	Intermediate	12	33.17	7.73	
	Secondary	79	33.91	5.61	
	Bachelors and above	296	33.05	6.07	

In this study, we compared the scores of TOPICOP and its sub-categories between two groups of participants: those who had experienced dermatological issues and those who had not. The results showed that participants without dermatological diseases had higher Global TOPICOP scores (33.88 ± 6.3) compared to those who had dermatological diseases (32.58 ± 5.7) (p=0.033). However, participants with dermatological diseases scored higher in the behaviors sub-category (p=0.019). Additionally, participants who had no dermatological issues were more likely to agree with the statement, "I do not know of any side effects, but I am still afraid of topical steroids" (Tables 6-7).

**Table 6:** Topical corticosteroid phobia (TOPICOP) scale questionnaire's results (N=389). Only previous users of topical corticosteroids were included (N=389). P-value for chi-square test (<0.05 is considered statistically significant).

	Suffered from dermatological disease	Totally disagree	Not really agree	Almost agree	Totally agree	P-value
Topical steroids pass into the bloodstream	No	13	52	88	37	0.168
		6.8%	27.4%	46.3%	19.5%	
	Yes	5	55	105	34	
		2.5%	27.6%	52.8%	17.1%	
Topical steroids can lead to infections	No	27	108	42	13	0.863
		14.2%	56.8%	22.1%	6.8%	
	Yes	26	117	46	10	
		13.1%	58.8%	23.1%	5.0%	
Topical steroids make you fat	No	27	108	42	13	0.112
		14.2%	56.8%	22.1%	6.8%	
	Yes	26	117	46	10	
		13.1%	58.8%	23.1%	5.0%	
Topical steroids damage your skin	No	8	56	100	26	0.865
		4.2%	29.5%	52.6%	13.7%	
	Yes	8	52	113	26	
		4.0%	26.1%	56.8%	13.1%	
Topical steroids will affect my future health	No	8	60	82	40	0.583
		4.2%	31.6%	43.2%	21.1%	
	Yes	10	59	97	33	
		5.0%	29.6%	48.7%	16.6%	
Topical steroids can lead to asthma	No	35	108	38	9	0.343
		18.4%	56.8%	20.0%	4.7%	
	Yes	39	125	30	5	
		19.6%	62.8%	15.1%	2.5%	
I don’t know of any side effects but I’m still afraid of topical steroids	No	17	45	83	45	0.026
		8.9%	23.7%	43.7%	23.7%	
	Yes	28	66	73	32	
		14.1%	33.2%	36.7%	16.1%	
I’m afraid of applying too much cream	No	7	33	53	97	0.837
		3.7%	17.4%	27.9%	51.1%	
	Yes	5	40	54	100	
		2.5%	20.1%	27.1%	50.3%	
i’m afraid of putting cream on certain zones like eyelids, where the skin is thinner	No	4	17	38	131	0.741
		2.1%	8.9%	20.0%	68.9%	
	Yes	7	21	35	136	
		3.5%	10.6%	17.6%	68.3%	
I wait as long as I can before treating myself	No	28	41	56	65	0.121
		14.7%	21.6%	29.5%	34.2%	
	Yes	38	52	62	47	
		19.1%	26.1%	31.2%	23.6%	

**Table 7:** Comparison of TOPICOP scale’s subcategory scores between participants with and without dermatological diseases (N=389). Analysis includes only previous users of topical corticosteroids (N=389). P-value obtained from student's t-test (<0.05 is considered statistically significant). The Knowledge and Beliefs subcategory scores range from a minimum of 6 to a maximum of 24. The Fears subcategory scores range from a minimum of 3 to a maximum of 12. The Behaviors subcategory scores range from a minimum of 3 to a maximum of 12. The Global TOPICOP score ranges from a minimum of 12 to a maximum of 48. TOPICOP: Topical Corticosteroid Phobia.

Subcategory	Suffer from dermatological issues	N	Mean	SD	P-value
Knowledge and beliefs	No	190	15.20	3.33	0.230
	Yes	199	14.82	2.83	
Fears	No	190	9.64	1.92	0.080
	Yes	199	9.31	1.85	
Behaviors	No	190	9.04	2.38	0.019
	Yes	199	8.45	2.56	
Global TOPICOP score	No	190	33.88	6.32	0.033
	Yes	199	32.58	5.70	

## Discussion

Successful treatment with topical corticosteroids requires meticulous attention to various patient and medication-related factors by a specialist. However, misinformation about possible adverse effects can contribute to a growing fear of these medications. These fears might result in suboptimal compliance and unsatisfactory therapeutic outcomes. We conducted a study to assess the knowledge and attitudes of previous users towards topical corticosteroids in Saudi Arabia. We used a validated online questionnaire and received responses from 397 individuals. Most respondents were Saudi citizens, females, aged 18-25 years, and held a bachelor's degree or higher. The findings provide valuable insights into the understanding and perception of topical corticosteroids among different demographics and highlight areas where educational interventions may be needed.

When assessing our demographic of previous users, there were alarming results regarding inappropriate indications. Out of 397 participants, 11 of them used topical corticosteroids as skin moisturizers, 19 used them as chemical peels, 39 used them for whitening purposes, and five used them to treat rosacea. 'Burns' was selected as an indication by 11 responders despite the paucity of conclusive evidence on the outcomes of topical corticosteroids in treating inflammation and hypergranulation of burns in the literature [8]. Included in the inappropriate indications selected were 'Skin Infections' and 'Fungal Infections'. When used alone, topical corticosteroids can exacerbate fungal infections, and long-term use might lead to secondary infections. A study where awareness and misuse among patients, pharmacists, and general practitioners were evaluated reported that the most common incorrect indication was superficial dermatophytosis in 89%, and the most prevalent adverse effect was recurrence or an increase in the extent of the infection in 76% of patients [9].

Many respondents could identify correct information regarding the different formulations of topical corticosteroids and their appropriate uses. For example, 80.2% of respondents knew that high-potency topical corticosteroids should not be used continuously for over three weeks. However, critical misconceptions persist, particularly regarding the side effects of topical corticosteroids. For instance, only 38.6% of respondents recognized increased hair growth, and 35.5% identified delayed wound healing as potential side effects. Additionally, 72.2% believed side effects were irreversible. This lack of understanding is reflected in both local and international studies; a nationwide South Korean study revealed that over half of the participants were unaware of the side effects of topical corticosteroids, and 1% believed there were none. One-fourth of the participants agreed that topical corticosteroids should be used until symptoms resolve, irrespective of the duration [10]. Similarly, a Saudi cross-sectional study found that 61.3% of its participants were unaware of the side effects resulting from the misuse of topical corticosteroids on the face [11].

The study further reveals a notable gender disparity in knowledge, with female participants exhibiting significantly higher scores than their male counterparts. Likewise, gender-based differences in knowledge have been reported in previous local studies, as 16.0% of females were able to identify acne, photosensitivity, purpura, hirsutism, and skin pigmentation as adverse effects of topical corticosteroids, compared to only 4.2% of males [12]. This discrepancy may indicate gender differences in health literacy and engagement in dermatologic care in our population, which warrants further exploration.

When comparing those with dermatological diseases to those without, participants with dermatological diseases displayed a better understanding of specific side effects of topical corticosteroids, such as increased skin vessel visibility. This insight likely stems from their personal experiences or targeted education during clinical encounters. In contrast, those without skin issues demonstrated a higher rate of misconceptions, underscoring the importance of extending educational efforts beyond clinical settings.

The discrepancy in the source of knowledge among responders is also concerning; those with dermatological diseases cited physicians as their primary information source more frequently, whereas individuals without such conditions were more likely to rely on pharmacists, family, friends, and social media outlets. This dependency on less reliable sources or less specialized healthcare professionals could contribute to the spread of misinformation. A study conducted in the United Kingdom found that more than 60% of pharmacists had incorrect knowledge about the different categories of topical corticosteroid potency. Additionally, 75% of pharmacists believed that patients do not need to be informed about the potency of their topical corticosteroids [13]. Similarly, only 1% of general practitioners in a Moroccan study could identify all side effects of topical corticosteroids [14].

The TOPICOP scale assessment revealed a significant prevalence of topical corticosteroid phobia. Interestingly, younger participants aged 18 to 25 years had lower phobia scores compared to participants aged 56 years and above. This finding might be due to long-standing misconceptions or a lack of exposure to updated medical information in older participants, contrasting previous studies that found no association with age [15-17]. Other characteristics of our population, such as gender, nationality, region, and educational level, did not show any statistically significant differences in phobia scores.

The average prevalence of topical corticosteroid phobia in patients with dermatological conditions, as seen in studies that exclusively included patients with all dermatological conditions, was 51.0% [18]. However, in our study, when comparing those with dermatological diseases to those without, participants without skin issues had higher overall TOPICOP scores. Despite their lack of knowledge regarding specific side effects, they were often apprehensive about topical corticosteroids. Nonetheless, participants with dermatological conditions scored higher in the behaviors sub-category, as demonstrated in two questions: 'I wait as long as I can before treating myself' and 'I stop the treatment as soon as I can'.

This study has some limitations. Firstly, the cross-sectional nature and reliance on self-reported data might introduce recall bias. Secondly, the use of convenience sampling limits the generalizability of our findings. Finally, participants were not instructed that responses should be made for topical corticosteroid single agents only. This may affect the selection of skin infections, fungal infections, and skin whitening as appropriate indications, as topical steroids can be used in conjunction with anti-infectious and lightening agents for their anti-inflammatory properties. Nevertheless, this study's strengths include being the first nationwide effort to assess knowledge and corticophobia in individuals with a history of topical corticosteroid use, using detailed and validated questionnaires.

## Conclusions

In conclusion, this study uncovers multiple areas of knowledge gaps about topical corticosteroids among previous users in the general population of Saudi Arabia, such as their proper indications and side effects. Improper indications among participants included moisturization, chemical peeling, whitening purposes, and rosacea. Moreover, female participants and individuals with dermatological diseases exhibited superior knowledge of the side effects of topical corticosteroids. Participants with dermatological diseases identified physicians as their primary source of information, while others relied more on pharmacists, family, friends, and social media platforms. Phobia towards topical corticosteroids was more prevalent among older participants aged 56 years and above and those without a history of dermatological conditions. Those with dermatological conditions scored higher in questions related to behaviors. A better understanding of the indications, proper uses, and possible complications of topical corticosteroids could reduce corticophobia and improve compliance. Therefore, extending educational efforts by dermatologists towards vulnerable groups, such as males and older patients, beyond clinical settings is recommended.
